# An analysis of the impact of pre‐analytical factors on the urine proteome: Sample processing time, temperature, and proteolysis

**DOI:** 10.1002/prca.201400079

**Published:** 2015-02-26

**Authors:** Sophie Hepburn, David A. Cairns, David Jackson, Rachel A. Craven, Beverley Riley, Michelle Hutchinson, Steven Wood, Matthew Welberry Smith, Douglas Thompson, Rosamonde E. Banks

**Affiliations:** ^1^ Clinical and Biomedical Proteomics Group, Leeds Institute of Cancer and PathologySt James's University HospitalLeedsUK; ^2^ Department of Blood SciencesThe General InfirmaryLeedsUK; ^3^ Nonlinear DynamicsNewcastleUK; ^4^ Department of Renal MedicineSt James's University HospitalLeedsUK

**Keywords:** Peptides, Protease inhibitor, Proteomics, Temperature, Urine

## Abstract

**Purpose:**

We have examined the impact of sample processing time delay, temperature, and the addition of protease inhibitors (PIs) on the urinary proteome and peptidome, an important aspect of biomarker studies.

**Experimental design:**

Ten urine samples from patients with varying pathologies were each divided and PIs added to one‐half, with aliquots of each then processed and frozen immediately, or after a delay of 6 h at 4°C or room temperature (20–22°C), effectively yielding 60 samples in total. Samples were then analyzed by 2D‐PAGE, SELDI‐TOF‐MS, and immunoassay.

**Results:**

Interindividual variability in profiles was the dominant feature in all analyses. Minimal changes were observed by 2D‐PAGE as a result of delay in processing, temperature, or PIs and no changes were seen in IgG, albumin, β_2_‐microglobulin, or α_1_‐microglobulin measured by immunoassay. Analysis of peptides showed clustering of some samples by presence/absence of PIs but the extent was very patient‐dependent with most samples showing minimal effects.

**Conclusions and clinical relevance:**

The extent of processing‐induced changes and the benefit of PI addition are patient‐ and sample‐dependent. A consistent processing methodology is essential within a study to avoid any confounding of the results.

**Abbreviations**
PI(s): protease inhibitor(s)
RT: room temperature

## 1 Introduction

There is considerable interest in the use of urine for biomarker studies, particularly in urological diseases since it potentially contains proteins secreted or shed directly from the kidney, bladder, or prostate at higher concentrations than in the peripheral circulation, in addition to products of glomerular filtration from the systemic circulation. Additionally, it can be collected noninvasively in relatively large quantities, is less complex than serum, repeated sampling is possible for monitoring, and the majority of proteins are soluble 1, 2, 3. However, analytical challenges include its dilute nature and high salt content, and its marked biological variability, being influenced by a variety of factors including hydration state, diet, timing, exercise, gender, and age 1, 2, 3.

**Clinical Relevance**

Urine is often used for biomarker discovery studies in diseases affecting the urinary tract. However, relatively few studies have investigated the potential impact of pre‐analytical factors on urinary proteins and most such studies have used urine from healthy controls. This study has investigated the effects of processing time, temperature, and use of PIs on proteins and peptides in urine using 2D‐PAGE and SELDI, and importantly using urine samples from patients from different disease groups. Overall, our conclusions are that the dominant factors are the disease and interpatient differences but that changes in peptides (and to a lesser extent proteins) can occur to a limited and variable extent during sample processing/storage depending on the patient, particularly involving proteolytic activity. It is important that within studies, a consistent sample processing methodology is employed in order to allow robust unbiased conclusions to be drawn, and that the stability of any potential identified biomarkers for the specific processing conditions is checked.

Relatively few urinary biomarkers have been approved by the FDA with examples including nuclear matrix protein 22 (NMP22) and bladder tumor associated antigen (complement factor H related protein/complement factor H) for use in bladder cancer surveillance 4 and there is now an enhanced effort to use proteomic technologies to identify new biomarkers. Recent proteomic studies employing extensive fractionation describe more than 2300 proteins in urine 5, and >100 000 different peptides with at least 5000 occurring in >20% of patients in any disease group 6. Such datasets provide a valuable resource with many available online (linked from www.urineproteomics.org). The potential of these types of study is now beginning to be recognized, as illustrated by the CKD273 peptide classifier for type 2 diabetic nephropathy 7.

Several studies describe the development of standardized urine proteomic analysis methodologies for different platforms with a consideration of the various technical aspects (e.g. 8, 9, 10, 11, 12). However, although the potential impact of various pre‐analytical factors during clinical sample processing on proteomic studies is increasingly being recognized 13, relatively few systematic studies have been undertaken using urine. Initiatives such as the “Biospecimen Reporting for Improved Study Quality” 14 provide guidance generically for some of the aspects to consider recording with studies involving clinical samples and a review has highlighted some of the areas that require further examination for urinary‐based studies, including the effects of protease inhibitors (PI) and processing/storage conditions 15. Various studies have investigated such aspects, but these are limited in mostly having used samples from healthy controls, often from only one or two individuals and often only analyzing or reporting relatively gross readouts, for example numbers of proteins identified. With these caveats, studies using LC‐MS/MS have reported no significant effects of storage at room temperature (RT) for up to 24 h 16 and no impact of PI during frozen storage as assessed by either LC‐MS/MS or 2D‐PAGE 9, 12. Analysis of peptides by MALDI/SELDI has shown temperature‐dependent effects with changes if stored at 4°C compared with −20°C 11 or 6 h at 25°C but not 4°C 17, small differences between 4°C and RT but increasing at 72 h compared with 1 or 6 h 18, and a decrease in the number of peaks at RT, which was not seen in the presence of PI until time points greater than 2 h 19. Using CE‐MS, leaving urine samples at 4°C for 24 h or RT for 6 h did not alter the statistical spread of results or result in altered classification of the samples in the model 10.

In this study, we have systematically examined the effects of sample processing time, temperature, and the presence or absence of PI on urine samples, parameters highlighted as requiring further study to contribute to the evidence underlying recommendations for standardized protocols 15. Proteins and peptides were examined globally using 2D‐PAGE and SELDI‐TOF‐MS, respectively, in addition to immunoassay of the specific proteins, retinol‐binding protein, β_2_‐microglobulin (β_2_M), α_1_‐microglobulin (α_1_M), IgG, and albumin. Importantly, samples used were from patients with a spectrum of renal/urological conditions as different pathologies may impact potentially more than would be seen in urine samples from healthy controls. Such studies examining pre‐analytical factors are critically important in the interpretation of biomarker studies, both in terms of allowing comparisons between studies and in providing evidence to drive the development of standardized protocols.

## 2 Materials and methods

### 2.1 Materials

Materials were purchased from the following suppliers as indicated: CyDye™ Cy5 DIGE Fluor minimal dye, Immobiline™ DryStrip IPG strips (pH 3–10NL, 24 cm; GE Healthcare, Amersham, UK); CHAPS (Calbiochem, Middlesex, UK); all consumables associated with SELDI (Bio‐Rad Laboratories, Hemel Hempstead, UK); ACN LC‐MS grade (Fisher Scientific UK Ltd, Loughborough, UK); Complete Mini, EDTA‐free, PI cocktail tablets (Roche, Lewes, UK); acetic acid (VWR, Leicestershire, UK); urea (MP Biomedicals, Strasbourg, France); TFA (ThermoScientific, Horsham, UK); sequencing grade modified porcine trypsin (Promega, Southampton, UK). All other chemicals were obtained from Sigma‐Aldrich (Dorset, UK), and were of analytical grade or above. Milli‐Q water was used throughout.

### 2.2 Urine sample collection and processing

**Figure 1:**
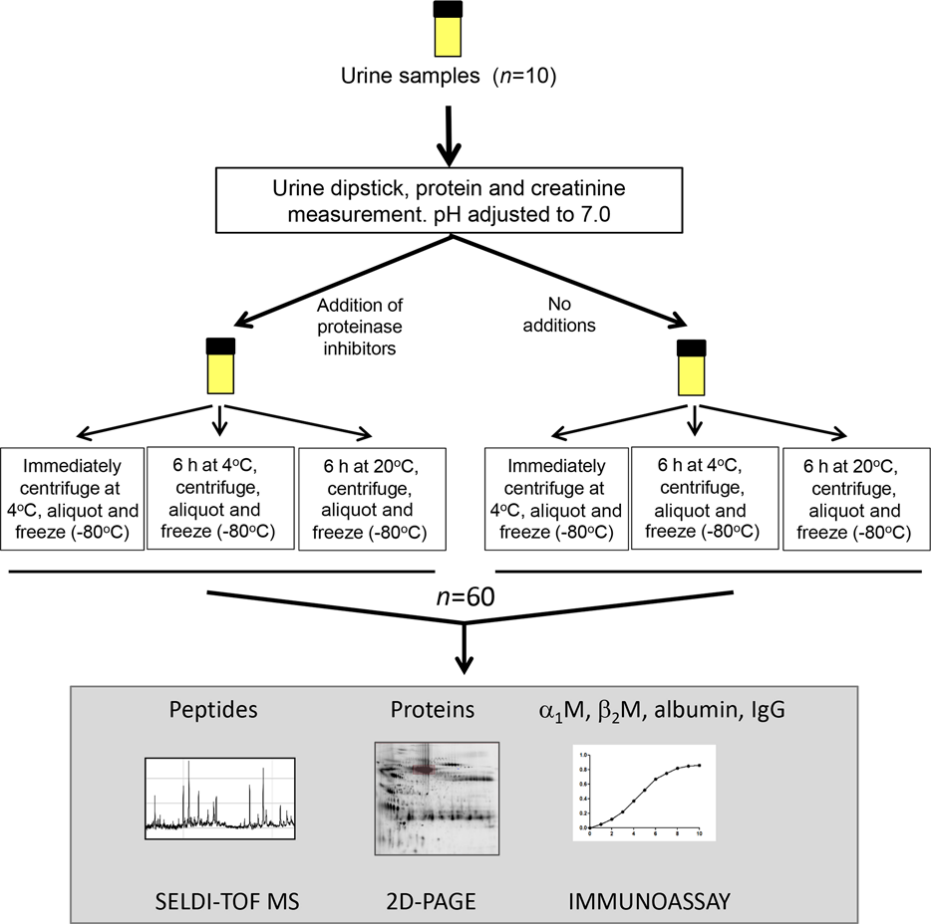
Schematic showing the study design. For immunoassay and SELDI, all ten samples each with six processing conditions were analyzed (for SELDI, 120 duplicate profiles were generated). For 2D‐PAGE, only six of the samples each with six processing conditions were used due to volume limitations and were analyzed in triplicate creating 108 gels.

Ten midstream urine samples were prospectively collected from nine patients following renal transplantation or attending St. James's University Hospital in Leeds with a variety of benign renal/urological conditions, between October 2009 and January 2010 (Table 1). The study was approved by the Leeds East Research Ethics committee and informed consent was obtained. Immediately following voiding, samples were tested for blood and protein using a dipstick (Siemens Multistix, Frimley, Surrey, UK), and an aliquot removed for protein and creatinine determination using a Siemens Advia 1800 analyzer (Siemens) in the Blood Sciences Department, Leeds General Infirmary. After adjusting the pH to 7.0, samples were each divided into two and PI was added to one‐half (one tablet per 25 mL urine). These two aliquots of each sample were then further subdivided as indicated (Fig. 1) to investigate the effects of processing times (immediate vs. 6 h delay) and temperature during the delay period (4°C vs. RT (in this case 20–22°C), prior to centrifugation at 2000 × *g* for 10 min, and removal and storage of the urine supernatant at −80°C until analysis.

**Table 1:** Patient characteristics and urine biochemistry

Patient	Gender	Age	Clinical scenario	Creatinine	Protein	PCR	pH	Blood	α_1_M	β_2_M	Albumin	IgG
	(M/F)	(years)		(mmol/L)	(mg/mL)				(mg/L)	(mg/L)	(mg/L)	(mg/L)
Aa)	M	31	19 days post‐RTx	3.7	0.13	35.1	5.19	+++	20	0.1	43	7
B	M	49	29 days post‐RTx	8.0	3.45	431.3	4.97	+	74	0.9	2873	114
C	M	42	8 days post‐RTx	1.6	0.04	25.0	6.48	Neg	11	2.2	<3	2
Da)	M	31	26 days post‐RTx	10.6	0.61	57.5	5.31	+++	54	0.3	322	39
E	M	56	12 days post‐RTx	5.6	0.20	35.7	5.50	+	26	0.9	70	12
F	M	48	Renal stones	3.6	0.16	44.4	7.25	Neg	8	0.1	<20	5
G	M	62	Renal stones	6.6	0.70	106.1	6.23	Neg	11	0.5	478	48
H	F	48	Renal stones	3.1	0.03	9.7	5.62	Neg	6	<0.1	<20	2
I	F	71	Recurrent UTIs	4.8	0.19	39.6	5.80	Trace	17	1.5	36	17
J	F	50	Recurrent UTIs	3.8	0.04	10.5	5.64	Neg	<6	<0.1	<20	7

α_1_M, α_1_ microglobulin; β_2_M, β_2_ microglobulin; Neg, negative; PCR, protein creatinine ratio; RTx, renal transplantation; UTI, urinary tract infection; blood, based on dipstick reading.

a) The samples labeled as patients A and D originated from the same patient 1 wk apart.

### 2.3 Proteomic analysis

#### 2.3.1 Sample concentration for analysis

Urine samples were thawed at RT and concentrated by centrifugal filtration according to the manufacturer's instructions and with starting volumes depending on the protein concentration. For analysis by 2D‐PAGE, 0.5–6.5 mL of each sample was concentrated using Amicon 10 kDa MWCO Ultra‐15 filtration units with the filtrate (150–200 μL), vacuum‐centrifuged for 1.5 h, and the resulting pellet was resuspended in DIGE lysis buffer (7 M urea, 2 M thiourea, and 4% w/v CHAPS). For analysis by SELDI, 0.1–1 mL of each sample was concentrated using Amicon® 3 kDa MWCO Ultra 0.5 mL centrifugal devices to a final volume of 100–200 μL. Protein concentrations were determined using the Bradford assay (Bio‐Rad Laboratories).

#### 2.3.2 SELDI‐TOF‐MS

Peptide profiling of the 60 urine samples was undertaken using a SELDI‐TOF mass spectrometer (ProteinChip® System, series 4000, Ciphergen, Fremont, CA, USA) and Ciphergen Express™ Client software (v3.0) for data analysis. IMAC30‐Cu (immobilized metal affinity capture array with copper surface) ProteinChip™ arrays were selected for use following initial studies, and urine sample and matrix application to ProteinChip arrays and data collection were performed as previously 20, 21. A QC sample was prepared by pooling 3 μg of all 60 urine samples. All samples were analyzed blind in triplicate and block randomized within a patient, with one spot on each sample chip containing the pooled QC sample. Following data extraction, duplicate profiles passing QC were selected for each sample analysis (with a fail rate of ∼20–30%, this strategy minimized reruns with analysis in duplicate being our normal practice). External mass calibration was performed with a standard calibration mixture of peptides and proteins from Ciphergen (three‐parameter weighted): bovine insulin β‐chain (3495.9 Da), human insulin (5807.6 Da), and recombinant hirudin (6963.5 Da). Peaks within the range 3–10 kDa were detected using a signal/noise threshold of 2.0 (centroid fraction 10.0%), and profiles examined for peak number and redundancy within each urine sample exposed to various processing protocols.

#### 2.3.3 2D‐PAGE and protein identification

Samples A, B, D, E, G, and I (the remaining samples had insufficient volume/protein) were adjusted to 1.0 mg/mL protein concentration and labeled individually in triplicate with Cy5 (CyDye DIGE Fluor, minimal dye) as previously described 22. Labeled samples (50 μg per strip) were separated by IEF using 24‐cm pH 3–10NL Immobiline IPG strips prior to electrophoresis in the second dimension on 24‐cm 10% polyacrylamide gels and fluorescence imaging. The major central forms of albumin were allowed to saturate in scanning, to improve the detection of lower abundance species, and excluded from downstream analysis; scans were normalized to the next most intense species in each image. Gel images were analyzed using Progenesis SameSpots software v4.5 (TotalLab, Newcastle‐upon‐Tyne, UK). QC analysis was first carried out on a run‐by‐run basis incorporating automated image assessment in the “Image QC” module combined with further multivariate analyses and visual inspection. Runs failing QC metrics were repeated and appropriate gels were selected for subsequent quantitative analyses. These were done separately for each sample, with the six handling conditions being compared in triplicate. Cropping, automatic reference gel selection, alignment, and spot detection were carried out, with manual correction where necessary. Spot normalized volume data were then exported in .csv (comma‐separated values) format for statistical analysis. To allow the different patterns to be related qualitatively for conservation of changes, a PG240 (TotalLab) experiment containing all the master reference maps was generated from the SameSpots archives and used to cross‐match all the patterns.

#### 2.3.4 Immunoassay of specific urinary proteins

The specific urinary proteins, β_2_‐microglobulin (β_2_M), α_1_‐microglobulin (α_1_M), IgG, and albumin, were determined by the Protein Reference Unit at Sheffield Teaching Hospitals. Briefly, β_2_M, IgG, and albumin were measured by immunoturbidimetry (Cobas Core, Roche Diagnostics, Burgess Hill, UK), and α_1_M by immunonephelometry using the Behring Nephelometric Analyzer II (Siemens). The LOD (and CV) for the specific protein assays was as follows: α_1_M 2.0 mg/L (7%), β_2_M 0.2 mg/L (5%), albumin 3 mg/L (5%), and IgG 4 mg/L (8%).

### 2.4 Statistical analysis

The SELDI and gel data were examined by PCA and hierarchical clustering analysis using a Euclidean distance metric and Ward's agglomeration method, for all data combined and separately for each urine sample condition.

The effect of PI addition and processing temperature/delay on detected peaks from the SELDI‐TOF spectra was evaluated using linear mixed effects model when considering multiple patients and linear models when considering individual patients. The linear mixed effect model can be represented through the equation:

y ijkt =α+βxi+γzt+δxizt+ηj+ɛ ijkt

A separate model was fitted for each peak, and separate models were fitted for all samples, post‐transplant samples, and benign urological disease samples. In the model for all samples, the response variable y_ijtk_ is the intensity value at one peak cluster. Here, *i* indexes the inclusion of PI (+PI—reference level compared with −PI), *j* the subject, *k* the repeat number (since all combinations were run in duplicate), and *t* the processing temperature (−80°C—reference level compared with 4°C and RT). An interaction term in the model for PI inclusion and processing temperature was also included, that is a term that will indicate a departure from additive effects in the model. These are the fixed effects in the model.

The random effect η_j_ in the model (η_j_ ∼ N(0,σ_η_^2^)) describes a subject‐specific effect and allows for the correlation between peak intensities measured on the same subject. The final term describes the residual variation (ε_ijkt_ ∼ N(0,σ_ε_^2^)), assumed to be uncorrelated with the other terms.

For each sample individually, linear models were considered. These models were fitted separately for each peak and each sample and can be represented through the equation:

y ikt =α′+β′xi+γ′zt+δ′xizt+ηj+ɛ ikt

As above, *i* indexes the inclusion of PI (+PI, reference level compared with −PI), *k* the repeat number, and *t* the processing temperature (−80°C, reference level compared with 4°C and RT). An interaction term in the model for PI inclusion and processing temperature was also included, again to indicate any departure from additive effects in the model. When examining fixed effects from the linear mixed effects and linear models, significance levels of 0.1% (*p* < 0.001) were considered significant as an ad hoc measure to control the false discovery rate. All analyses were undertaken in the R environment for statistical computing (R Development Core Team, Vienna) using the lmer() function in library(lme4) for the linear mixed effects models 23 and lm() for the linear models.

For the gels, linear models were employed to generate three key datasets: (i) alterations within sample associated with PI, independent of/across temperatures; (ii) alterations within sample associated with temperature, independent of PI status; and (iii) alterations within sample associated with PI but showed a differing effect of PI across temperatures. In the first two cases, simple cut‐offs (≥2‐fold elevation or decrease in the linear model, *p* ≤ 0.05) were applied to select spots for identification. In the third case, the target was spots that were altered by PI more notably at higher temperatures, so searching for degradation under harsher conditions that PI treatment opposed. This required a heterogeneity (*p* ≤ 0.05) across temperature groups for PI effect, and a typical (≥2‐fold elevation or decrease in the linear model, *p* ≤ 0.05) cut‐off combination at RT, the group that would be expected to be most affected. Furthermore, a simple requirement of increasing divergence in mean fold change terms of −80°C < 4°C < RT was applied.

## 3 Results

### 3.1 Patient characteristics

Brief characteristics of the patients and their urinalysis results are shown in Table 1. Elevated results for many of the analytes are seen reflecting the different underlying disease processes and the intended heterogeneity of the samples used in this study.

### 3.2 Peptide and protein alterations

#### 3.2.1 Peptide profiles (SELDI‐TOF)

**Figure 2:**
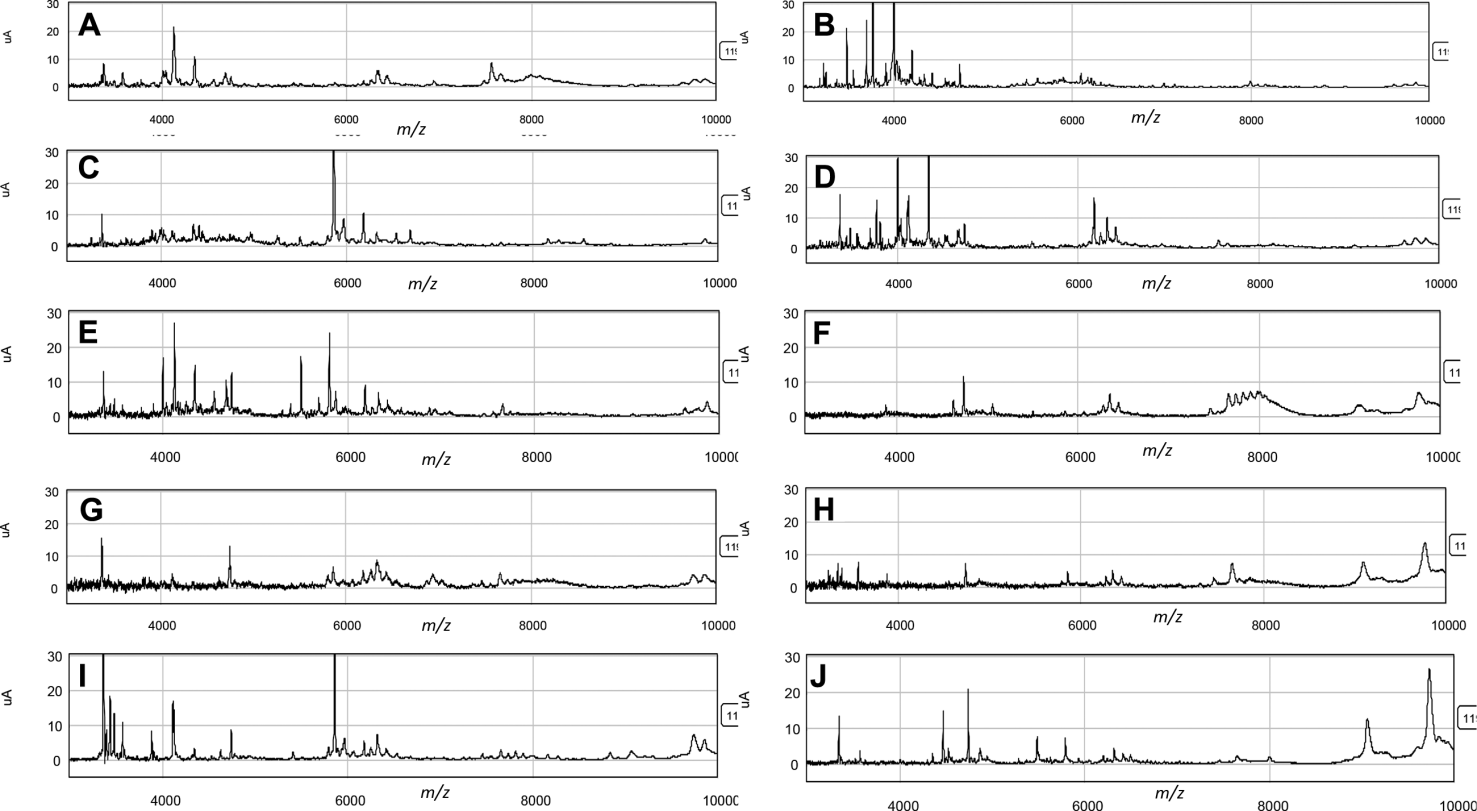
Representative examples of the SELDI profiles of each sample.

**Figure 3:**
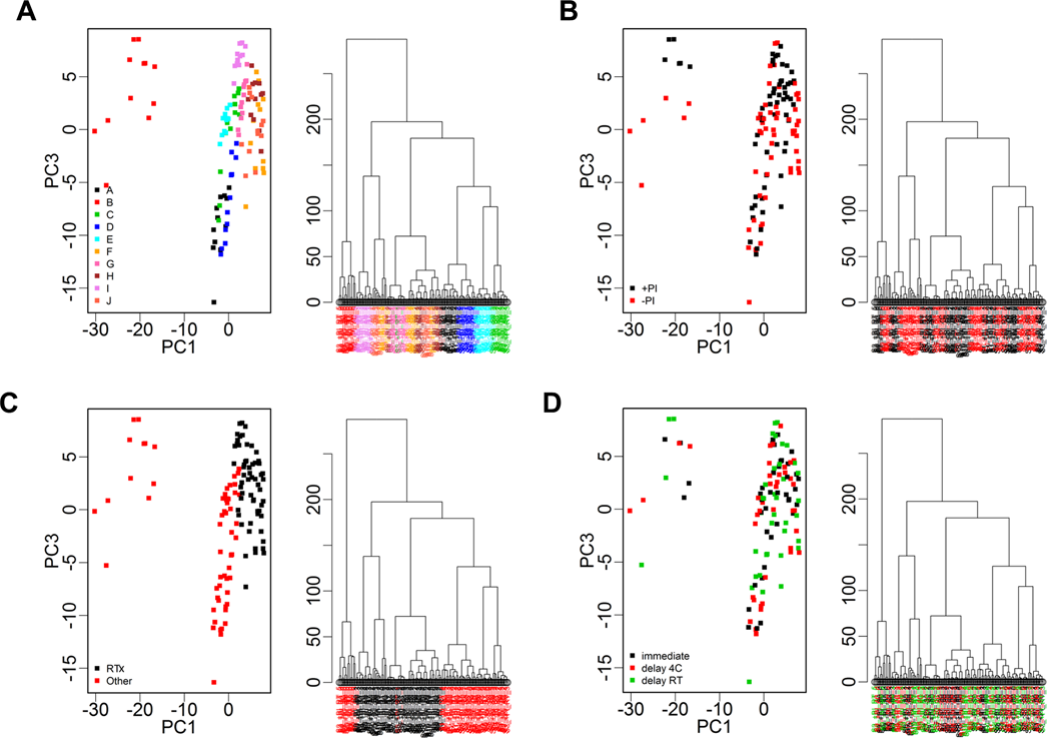
PCA (PC1 vs. PC3) and hierarchical clustering of proteomic profile obtained by SELDI considering all patients and replicate samples. Color coding represents (A) patient (A–J), (B) addition of protease inhibitor (+PI/−PI), (C) patient status (RTx = renal transplant or other), and (D) processing conditions (immediate processing and stored at −80°C, delayed processing and stored at 4°C, and delay and stored at room temperature (RT; 20–22°C)). Clustering is apparent by patient and also disease status with one patient (B) having a profile that could be considered quite different from the others. The clustering by patient is clearer than any separation by protease inhibitor or processing condition indicating patient heterogeneity is a more dominant factor than the manner in which a sample is processed.

**Figure 4:**
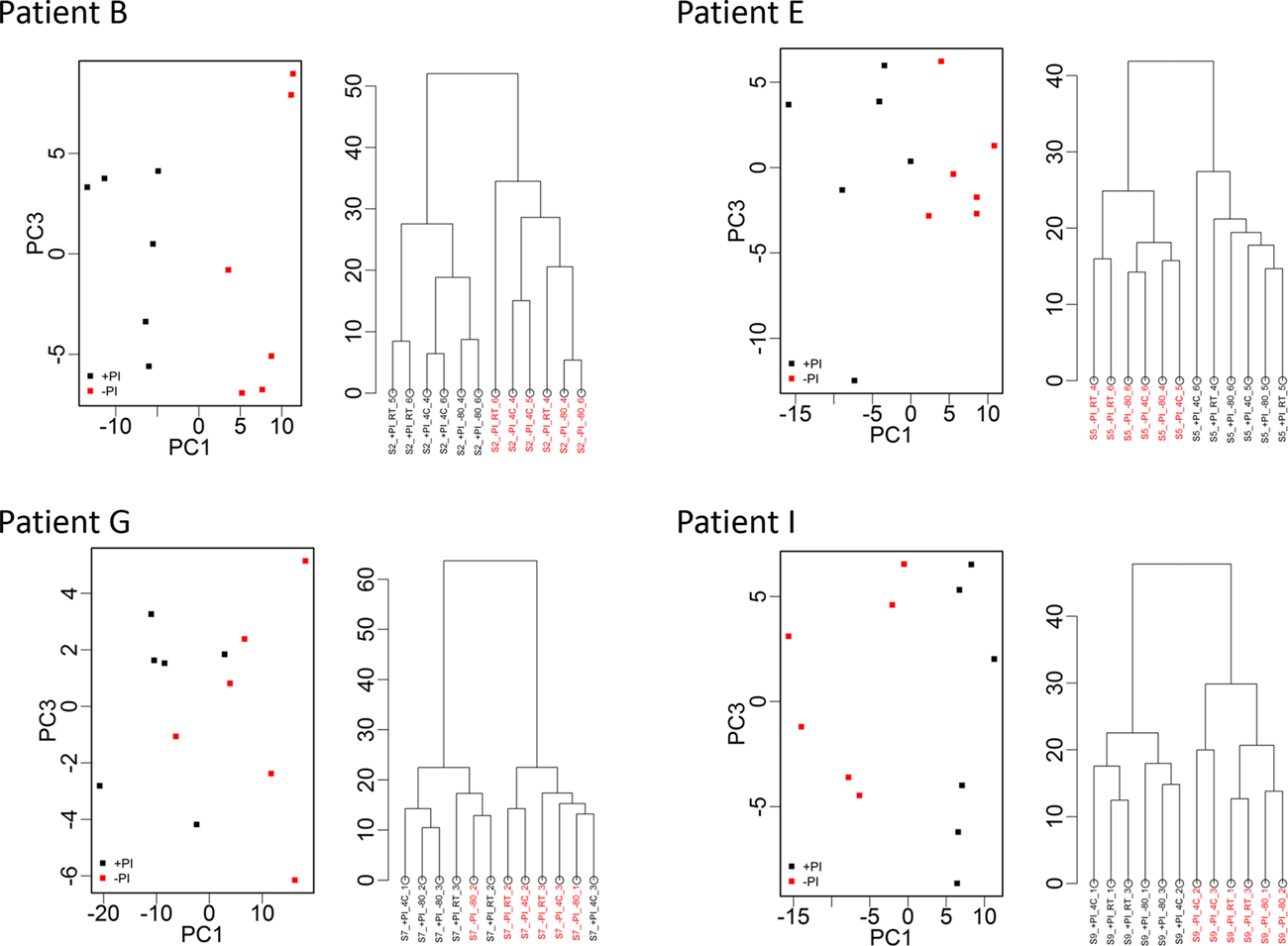
PCA (PC1 vs. PC3) and hierarchical clustering of proteomic profile obtained by SELDI considering samples from patients B, E, G, and I and replicate samples. Color coding represents addition of protease inhibitor (+PI/−PI) to a sample. There is clear separation in PCA and dendrograms by addition of protease inhibitor for three of the patients. Clear separation was not apparent when further examining plots related to other processing conditions.

The profiles of the samples from different patients differed markedly (Fig. 2). Overall, 228 peaks were detected across all samples combined with average peak numbers for different patients ranging from 156 (patient G) to 211 (patient A). Analysis of the CVs of all peaks in the pooled QC samples included on every chip showed a range of 23.5–109.4% with an overall median CV of 39.3%. PCA and hierarchical clustering analysis of data from all patient samples showed evidence of clustering predominantly by patient and also by broad clinical group (postrenal transplant or other; Fig. 3). No evidence of clustering on the basis of processing temperature/delay period or of technical analytical variables such as chip spot number was apparent, although some indication of clustering was seen on the basis of ±PI and when data from individual patients were examined, samples were perfectly separated by PI status in three of the ten patients (B, E, and I; Fig. 4).

**Figure 5:**
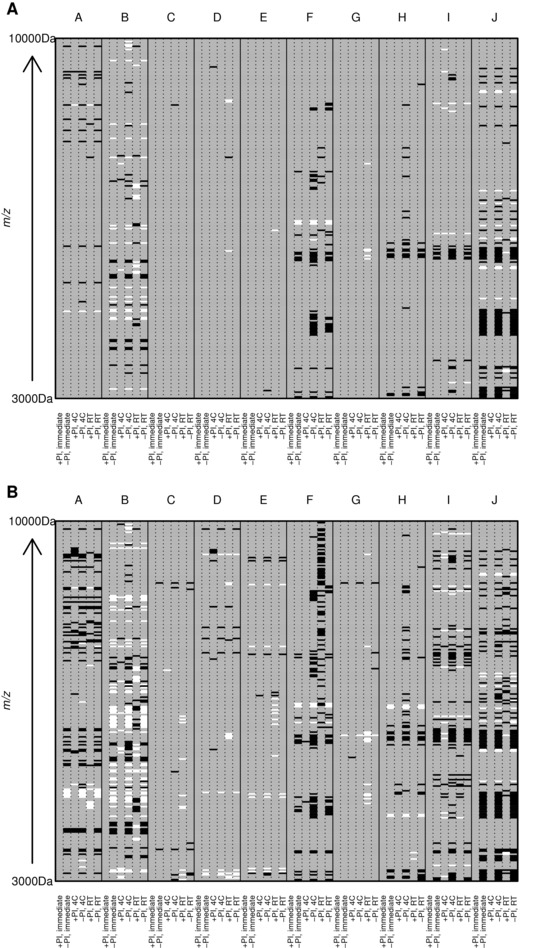
Heatmaps showing statistically significant changes in SELDI peak intensity from “gold standard” processing condition (+PI/immediate processing). Patients (A–J) are represented by columns with subcolumns representing each sample processing condition (−PI/immediate processing or ±PI delayed processing at 4°C or room temperature (20–22°C)). Rows represent SELDI peaks arranged by *m*/*z* ratio. Increases in peak intensity of (A) twofold or greater or (B) 1.5‐fold or greater, which are significant at *p* < 0.001, are represented by black rectangles and similarly significant decreases in peak intensity are represented by white rectangles. Peak *m*/*z* values are provided in Supporting Information Table 1 for the twofold changes.

Analysis of changes in specific peaks using lme and lm model results and using a cut‐off peak intensity of > 1, minimum fold difference of 2 (based on the technical variability), and *p* < 0.001 compared with the “gold standard” condition of +PI/immediate processing (assumed to have least proteolysis) confirmed the exploratory data analysis results mentioned above. For example, the most pronounced effect was seen in patient J where 56 of 182 (30.7%) peaks changed between the immediate processing/+PI and delayed processing for 6 h at RT/−PI (this patient also showed the biggest fold changes in specific peaks as can be seen in Supporting Information Fig. 1A and B where peaks with fold‐changes of 3 and 5 are shown, although few in number). This is in marked contrast to patients C, D, E, and G where nine peaks (1.2%) changed across all samples/conditions using the above criteria (Fig. 5A, Supporting Information Table 1). Interestingly, although samples from patient E clustered perfectly according to ±PI (Fig. 4), the changes seen did not reach the above criteria (Fig. 5A), although if a less stringent cut‐off of 1.5‐fold change was applied, the ±PI effect started to become more apparent (Fig. 5B). This can be explained by other factors potentially interacting with the PI effects in some patient samples (e.g. J) and also it must be remembered that the first three principal components only explain on average 71% of the variance.

Although there were overlaps in the peaks affected, no consistent pattern was apparent across patients with the exception of the region of 6000–6600 *m*/*z* where several peaks increased in the absence of PI in several patients (Fig. 5A and B, Supporting Information Table 1). Peak changes generally in the absence of PI were fairly consistent within a patient and almost irrespective of delay time and temperature, although immediate processing in the absence of PI prevented some of the changes in patients F and H. Even in the presence of PI, delay at RT did lead to some changes in the four patients who showed the most marked changes overall in the absence of PI (patients B, F, I, and J), although often affecting different peaks to those affected in the absence of PI, and this trend can be seen more obviously when relaxing the threshold of fold‐change to 1.5× (Fig. 5B). To check for the possibility that the presence of the PI chemicals themselves was causing any effect on the spectra independently of their biological action, three processed urine samples were spiked with PI and immediately analyzed and compared with the spectra from the same urine samples in the absence of PI. Using a similar cut‐off of twofold change and *p* < 0.001, no significant difference in any peaks was found in the presence and absence of PI (and only four peaks overall at a 1.5‐fold change) and no clustering on the basis of PI.

#### 3.2.2 Protein profiles (2D‐PAGE)

**Figure 6:**
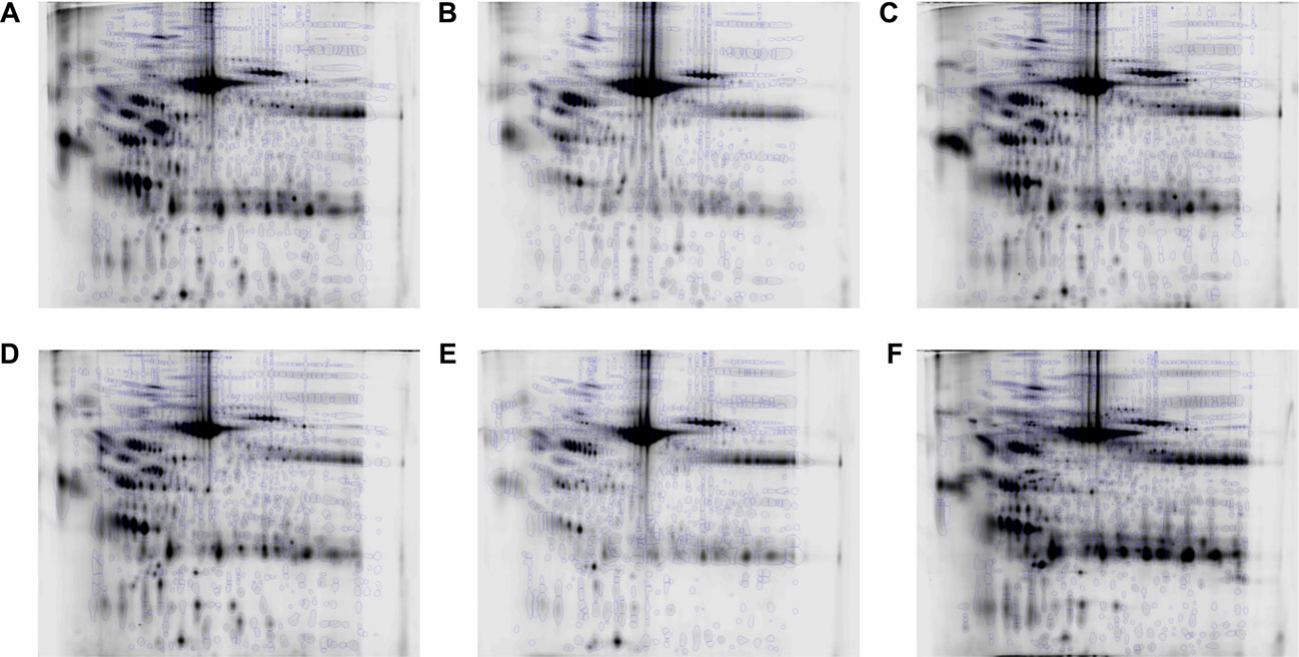
The reference image from each patient with the final spot pattern superimposed. (A) Patient A; (B) patient B; (C) patient D; (D) patient E; (E) patient G; (F) patient I.

**Figure 7:**
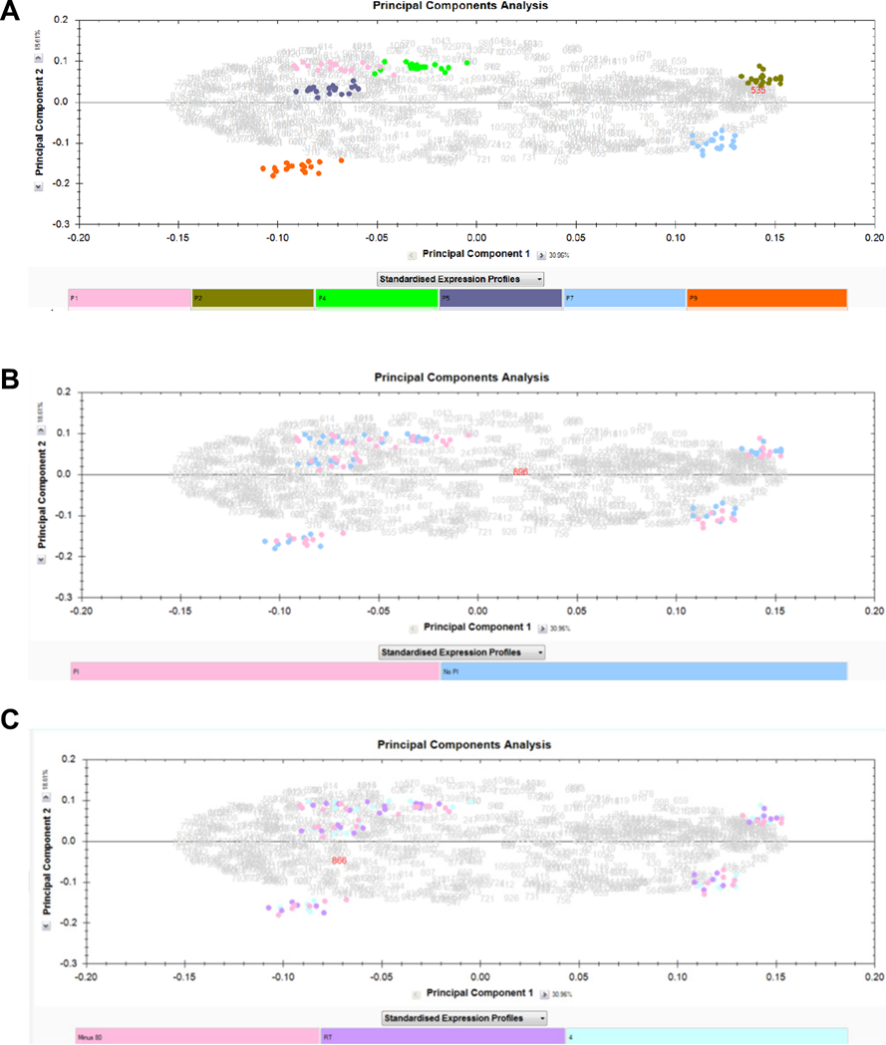
PCA of all 108 gels in a single SameSpots experiment, generated within the software and showing the first two eigenvectors in each case. The color key is below each plot. (A) All 108 samples colored by patient (pink = A, olive = B, green = D, mid‐blue = E, light blue = G, orange = I); (B) by PI status; (C) by temperature. The first two components cumulatively explain 49.57% of the total variance.

The numbers of spot features present in the final profile of each sample are provided in Table 2, and the reference image resulting from each analysis is shown with pattern overlaid in Fig. 6. The mean number of spots detected was 1082. Separate intrapatient analyses (with the repeat time points A and D for the same patient also being treated separately) were seen to be necessary to investigate processing effects. Although the profiles between patients were grossly similar (Fig. 6), especially the repeat‐sampled A and D, there were many differences in moderate to low abundance species between patients, meaning that a key assumption of 2DE analysis, that most species present remain unaltered, would have potentially been violated in a single analysis comparing all the samples. To illustrate this, such an analysis was additionally carried out, using the six aligned image sets and cross‐aligning them further via their reference gels using visually conserved proteins as landmarks, to align all 108 gels. Plotting the first two principal components in SameSpots showed that interpatient variation overwhelmingly dominated the separation with no comparable level of effect of PI status or temperature (Fig. 7). Indeed, 849 of 855 spots detected in this 108‐gel experiment were altered significantly across the six patient sample sets by ANOVA at the 0.05 level as reported by SameSpots. This supported the use of separate intrapatient analyses for the discovery of alterations associated with processing. Further supporting this, the serial samples from patient A/D (treated as two patients) showed similar behavior and a very similar number of spots were detected. Figure 7 shows that even repeat sampling and analysis of the same individual is associated with more variability than that caused by variation in processing method. Examination by PCA of individual patients on the basis of PI showed no apparent clustering.

**Table 2:** Results from the six 2D‐PAGE intrapatient analyses showing the numbers of features detected in the final profile of each feature map, and the numbers of statistically significant alterations with each variable investigated according to the criteria in Section 2.4

Alterations	Sample						Conservation, where examined
	Aa)	B	Da)	E	G	I	
		
Features in profile	1117	878	1118	1219	949	1209	
**Target (i)**							
PI elevated	9	3	2	18	9	30	
Conserved in at least three samples							0
Conserved in at least three different individuals							0
PI reduced	24	23	10	28	39	19	
Conserved in at least three samples							5
Conserved in at least three different individuals							4
PI total	33	26	12	46	48	49	
**Target (ii)**							
4°C elevated	2	19	7	15	5	4	
Conserved in at least three samples							0
Conserved in at least three different individuals							0
4°C reduced	2	3	2	16	1	12	
Conserved in at least three samples							0
Conserved in at least three different individuals							0
4°C total	4	22	9	31	6	16	
RT elevated	10	9	4	11	1	3	
Conserved in at least three samples							0
Conserved in at least three different individuals							0
RT reduced	12	6	9	23	2	13	
Conserved in at least three samples							1
Conserved in at least three different individuals							0
RT total	22	15	13	34	3	16	
Elevation, both temps	0	0	1	2	0	0	
Reduction, both temps	0	0	2	11	0	5	
**Target (i) and (ii) combination**							
PI and 4°C (any direction)	0	0	1	11	0	6	
PI and RT (any direction)	0	1	1	15	2	11	
**Target (iii)**							
PI versus non‐PI heterogeneity analysis—elevated with PI	0	0	0	2	0	0	None conserved across patients
PI versus non‐PI heterogeneity analysis—reduced with PI	0	0	2	5	10	1	None conserved across patients
PI versus non‐PI heterogeneity analysis—total	0	0	2	7	10	1	None conserved across patients

As standard these were a ≥2‐fold change in the linear model, with *p* ≤ 0.05. For the heterogeneity analyses, three criteria were applied in the second linear model. The heterogeneity *p* value was 0.05 or less, there was a significant change with PI in the RT group by normal cut‐offs, and the mean fold change rose in the order −80°C < 4°C < RT.

a) A and D are treated as two patients but represent two samples taken from the same patient 1 wk apart.

**Figure 8:**
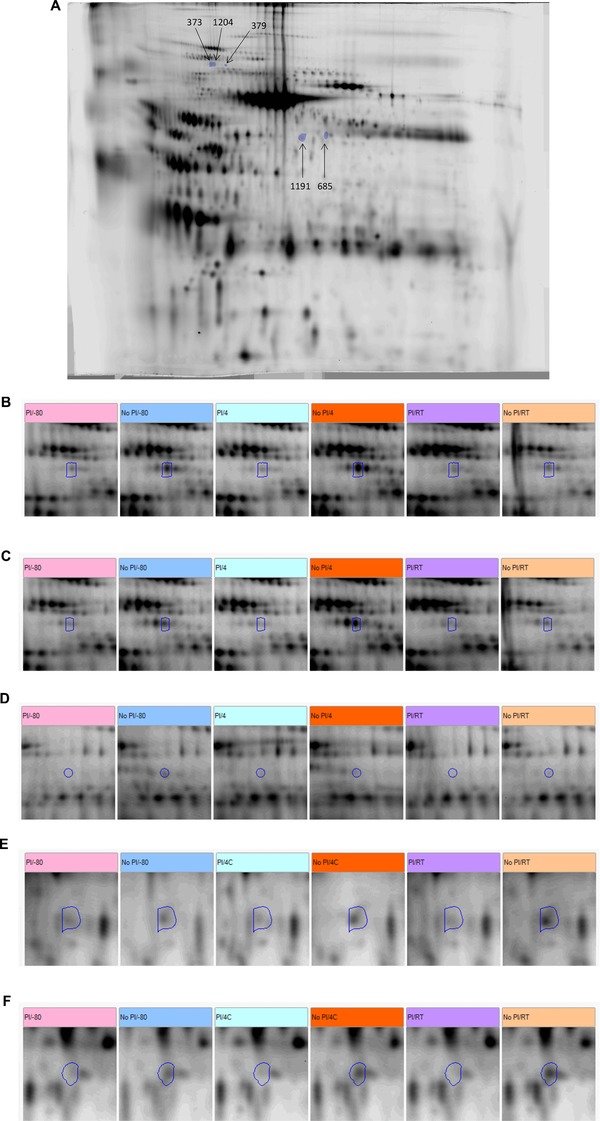
(A) The five “most conserved” changes altered with PI independently of temperature according to the standard cut‐offs in more than one sample. The numbers assigned are those from the master PG240 matching pattern with changes in spots 373, 379, 685, 1191, and 1204 being conserved in four, three, three, three, four samples, respectively, and all increased in the absence of PI. The image is of sample E at −80°C, +PI gel. (B)–(F) Features shown across the groups, in one gel from each condition of the samples that demonstrated an alteration in each feature, respectively: (B) 373 in E; (C) 1204 in E; (D) 379 in A; (E) 1191 in D; (F) 685 in D.

The numbers of proteins altered with processing within each sample by the criteria described in Section 2.4 are summarized in Table 2. While there were alterations with PI and temperature in each sample and more species appeared with the omission of PI than with its inclusion, the changes were very small in number, few were conserved, and any temperature‐associated alterations were quite different between 4°C and RT, at least at the cut‐offs used, which may reflect gradation. The five most conserved protein changes with PI are shown in Fig. 8. Attempts to identify the proteins associated with conserved PI‐induced changes were unsuccessful, presumably due to low abundance as they were not visible on preparatory gels. There were no conserved species altered across samples showing an increasing effect of PI with temperature.

#### 3.2.3 Specific proteins

Although there were marked differences in the concentrations of the specific proteins, β_2_M, α_1_M, IgG, and albumin, between samples from the different patients, no significant differences (*p* < 0.05) were detected between the different processing conditions (Supporting Information Table 2).

## 4 Discussion

Progress is being made in biomarker discovery studies through exploitation of the various technological developments in MS in particular, coupled with an increasing realization that the impact of pre‐analytical variables in clinical proteomic studies can be critical 13. Urine is increasingly being used in biomarker studies and even without superimposed pathological changes, its composition varies considerably physiologically due to factors such as diet, timing, exercise, gender, and age 1, 2, 3. This is also illustrated through a recent LC‐MS/MS analysis examining urine samples from seven individuals over 3 days where in >600 identified proteins, the technical variability with a median CV of 18% was far less than that of the intra‐ and interindividual variability at 48 and 66%, respectively 24. As discussed further below, our study also shows marked differences in protein and peptide profiles between samples from different individuals, even within similar disease groupings, illustrating the challenges of biomarker discovery in cross‐sectional comparisons where even “background” variability is so high. Although careful patient matching may help, it is likely that longitudinal comparisons of samples matched within patients, for example pre‐ and postremoval removal of a cancer, may overcome such background heterogeneity and lead to more easily identifiable disease‐related changes.

However, some of the technical pre‐analytical factors are less well investigated, in particular the evidence for whether proteolysis is an issue and addition of PI should be advocated, and the impact of different processing/storage conditions is still sparse 15. A tentative standard protocol for urine sample collection and processing has been proposed by the HUPO Human Kidney and Urine Proteome Project, for example recommending that PI addition may be necessary for proteinuric urine samples (with further evaluation planned), but not for normal urine samples (http://www.hkupp.org/Urine%20collectiion%20Documents.htm) and storage can be either at −20 or −80°C. The exception is where exosome analysis is involved when PI addition is advocated and this is also the case on the EuroProt website describing protocols for urinary exosome analysis (http://www3.niddk.nih.gov/intramural/UroProt/collection‐storage.shtml), together with storage at −80°C with significant reductions in recovery of exosomal proteins being reported after 7 months of storage at −20°C 25.

In our study, the changes in proteins as analyzed by 2D‐PAGE were insignificant in number and did not appear to be more prevalent in any particular set of conditions. This is also in agreement with most previous studies at the protein level, although some are quite limited in scope. Using SDS‐PAGE followed by LC‐MS/MS to analyze urine from a healthy control left at RT for 0, 4, 8, and 24 h, no significant differences were found in terms of the numbers of proteins detected or mean normalized spectral counts when the 200 most abundant proteins were examined in groups of 50 16. Similarly by iTRAQ, no significant changes relating to PI addition were seen in numbers or amounts of 83 proteins in urine samples from a healthy control, stored for <1 wk at −80°C ±PI 9. PI addition has also not been recommended as necessary based on 2D‐PAGE evaluation, but with the caveat that this was based on analysis of normal nonproteinuric urine samples stored at −30 or −70°C 12. In our study involving a variety of pathological urine samples, the number of changes seen in proteins following delays in sample processing at various temperatures was small and little effects of PI addition were seen by 2D‐PAGE. Clearly, some proteins were affected but careful validation of any findings relating to specific proteins should involve stability studies rather than necessarily supporting the addition of PI routinely. Examining specific proteins illustrates this, for example storage of urine samples at 4 or 25°C for 48 h prior to freezing versus immediate processing had no effect on NGAL, cystatin C, L‐FABP, or KIM‐1 but effects on IL‐18 were seen, particularly at 25°C, although whether this was due to proteolysis is not clear 26. Similarly, analysis of urine samples from pediatric diabetic patients stored at −20 versus −70°C for 6–8 months found creatinine to be stable but NAG, albumin, and retinol‐binding protein were underestimated following storage at −20°C, with marked interindividual variation and 36% of samples being substantially affected 27. Examination of albumin specifically, in various pathological urine samples stored for 12 months, also found marked degradation in 11 of 40 samples stored at −20°C compared with −70°C 28. Adjustment of the pH to 2.3–2.5 resulted in rapid albumin degradation consistent with pepsin‐type proteolytic activity and adjustment of pH to neutral and/or addition of PI was recommended 28.

Although not extensive and patient‐dependent (but consistent within individual patients), our study found more marked changes in peptides, probably reflecting the relative impacts of proteolysis with partial degradation of a protein spot being amplified in terms of the resultant peptide peaks detected against a low background. It is possible that some of the differences ±PI may have occurred not during the processing period per se as part of a continuous process but during subsequent storage at −80°C prior to analysis. In the case of SELDI, the period of storage was approximately 3 months and for the 2D PAGE analysis result, it was ∼2 years. However, we consider this to be the less likely explanation and this is supported partly by the slightly lower number of peaks affected in some of the immediately processed (∼30 min) samples without PI compared with those delayed for 6 h. Intuitively, as urine is stored in the bladder, much of the proteolysis would have expected to occur prior to voiding even, which cannot be prevented and in fact which presumably underlies the disease‐specific signatures of urinary peptides 7, 10, 29, but clearly our findings indicate that in some samples this may be an ongoing process, continuing following voiding. In the absence of PI (and even in the presence of PI at longer time delays and dependent on temperature), this may therefore be variable depending on processing conditions as indicated and potentially impact the results. The question of whether this supports addition of PI to samples though as routine must be considered against the various pros and cons. PI solutions/tablets are expensive, toxic, and can interfere with analysis, for example competing with proteins on SELDI chips depending on the surface chemistry (not the case in our study as shown in the spiking results) or binding to/introducing modifications to proteins as previously reported for 4‐(2‐aminoethyl) benzenesulfonyl fluoride 30. Depending on the model, continued and variable proteolysis in some samples may have no impact and may even just enhance the proteolytic differences underlying different diseases and classification models. Using CE‐MS and looking at 1200–2000 peptides per sample 10, derived models using 273 peptides 7 may not be affected and certainly many technical aspects have been investigated 7, 31 as part of ongoing studies. Stability analyses within CE‐MS studies examining a delay of 6 h at RT or 24 h at 4°C in three patient samples found CE‐MS results were not affected in terms of “statistical spread” or support vector machine (SVM) scores implying consistent classification 10, although no further details were provided about any effects seen on specific peaks. Similarly, storage of eight samples at 4°C for up to 2 wk prior to analysis was not found to adversely affect their classification in a diagnostic model 32.

Examining much smaller numbers of peptide peaks by MALDI/SELDI has shown some processing‐related effects with storage at 4°C prior to freezing resulting in small numbers of changes 11, 18 and changes in normal urine peptide profiles after 6 h at 25°C compared with 4°C 33 with marked interindividual variation. Interestingly, first‐void urine (but not midstream) showed considerable changes following 3 days storage at both 4°C and RT, with new peaks appearing in the 2–6 kDa range 34. A time‐dependent decrease in the number of urinary peaks in normal urine samples over 48 h at RT was prevented with the addition of PI, but only over the first 2 h with progressive changes being reported at later times even in the presence of PI 19. This is consistent with our observations using pathological urine samples, where those with the most marked changes in the absence of PI also started to show some changes after 6 h in the presence of PI, particularly at RT. The components of the commercial PI cocktail are proprietary but of the four major classes of proteolytic enzymes (serine, cysteine, metallo‐, and aspartic), it is aimed at inhibition of cysteine and serine proteases. Given that the pH of all samples was adjusted to neutral to avoid precipitation 35, it is unlikely that aspartic proteases that are active at acidic pH are responsible for any of the changes in peptides we have seen in the presence or absence of PI. However, the PI cocktail is EDTA‐free to avoid issues with the IMAC chemistry and therefore matrix‐metalloproteinases may account for some of the peptide changes. Certainly, examples of various matrix metalloproteinases and their complexes have been reported in urine in a variety of diseases including urinary tract infection, post‐renal transplantation with either acute/chronic rejection or fibrosis, and diabetes 36, 37, 38, 39, 40. The differences in peaks affected in the presence and absence of PI, although there is some overlap, are likely to reflect the differing patterns of activity/inhibition of the various contributing proteases and therefore different peptide masses. Unfortunately, the identities of the peptides could not be determined, which is a major limitation of the SELDI platform and which has precluded our modeling of possible proteases involved 29.

Control of analytical and pre‐analytical variables is crucial for the success of urine analysis, in order to obtain meaningful and reproducible data, even if the interpatient variability is difficult to avoid. The development of universal standard operating procedures for sample collection and handling is a complex task that will require shared experience and expertise, but this would better facilitate the future adoption of clinical proteomics into routine hospital practice.

Overall, our assessment of the impact of processing delays and addition of PIs to urine peptide and protein analysis has demonstrated that the dominant effects are the inter‐individual variability and underlying pathology. Processing‐related changes to proteins were minimal and although more marked, changes to peptides in the absence of PIs were very sample‐dependent, with many samples exhibiting no apparent degradation. We would advocate the use of PI where possible due to the unpredictability of proteolytic activity within samples but pragmatically, particularly for peptides, this must be balanced against the contributions of such activity to any diagnostic models. The most critical aspect is the adoption of consistent protocols within studies, their description within published studies, and the incorporation into any biomarker validation studies of investigations of potential impact of sample handling processes.

## Supporting information

As a service to our authors and readers, this journal provides supporting information supplied by the authors. Such materials are peer reviewed and may be re‐organized for online delivery, but are not copy‐edited or typeset. Technical support issues arising from supporting information (other than missing files) should be addressed to the authors.

**Figure S1**. Heatmaps showing statistically significant changes in SELDI peak intensity from “gold standard” processing condition (+PI/immediate processing). Patients (A‐J) are represented by columns with sub‐columns representing each sample processing condition (−PI/immediate processing or +/‐ PI delayed processing at 4°C or room temperature (20‐22°C)). Rows represent SELDI peaks arranged by m/z ratio. Increases in peak intensity of a). 3‐fold or greater or b). 5‐fold or greater, that are significant at P < 0.001 are represented by black rectangles and similarly significant decreases in peak intensity are represented by white rectangles.

**Table S1**. Urinary concentrations of α_1_‐microglobulin (α_1_M), β_2_‐microglobulin (β_2_M), albumin and IgG in 10 different urine samples (A to J) processed in the presence or absence of protease inhibitors either immediately or after delays of 6 hours at 20°C or 4°C.

**Table S2**.
