# Supplementary material for: An analysis of the impact of pre‐analytical factors on the urine proteome: Sample processing time, temperature, and proteolysis
Source: Proteomics Clin Appl. 2015 Feb 26;9(5-6):507–21. doi: 10.1002/prca.201400079 (PMC4964914; doi:10.1002/prca.201400079)
Supplement: Supplementary file 1 — Figure S1. Heatmaps showing statistically significant changes in SELDI peak intensity from “gold standard” processing condition (+PI/immediate processing). Patients (A‐J) are represented by columns with sub‐columns representing each sample processing condition (−PI/immediate processing or +/‐ PI delayed processing at 4°C or room temperature (20‐22°C)). Rows represent SELDI peaks arranged by m/z ratio. Increases in peak intensity of a). 3‐fold or greater or b). 5‐fold or greater, that are significant at P < 0.001 are represented by black rectangles and similarly significant decreases in peak intensity are represented by white rectangles. [file PRCA-9-507-s001.pptx]

## Slide 1
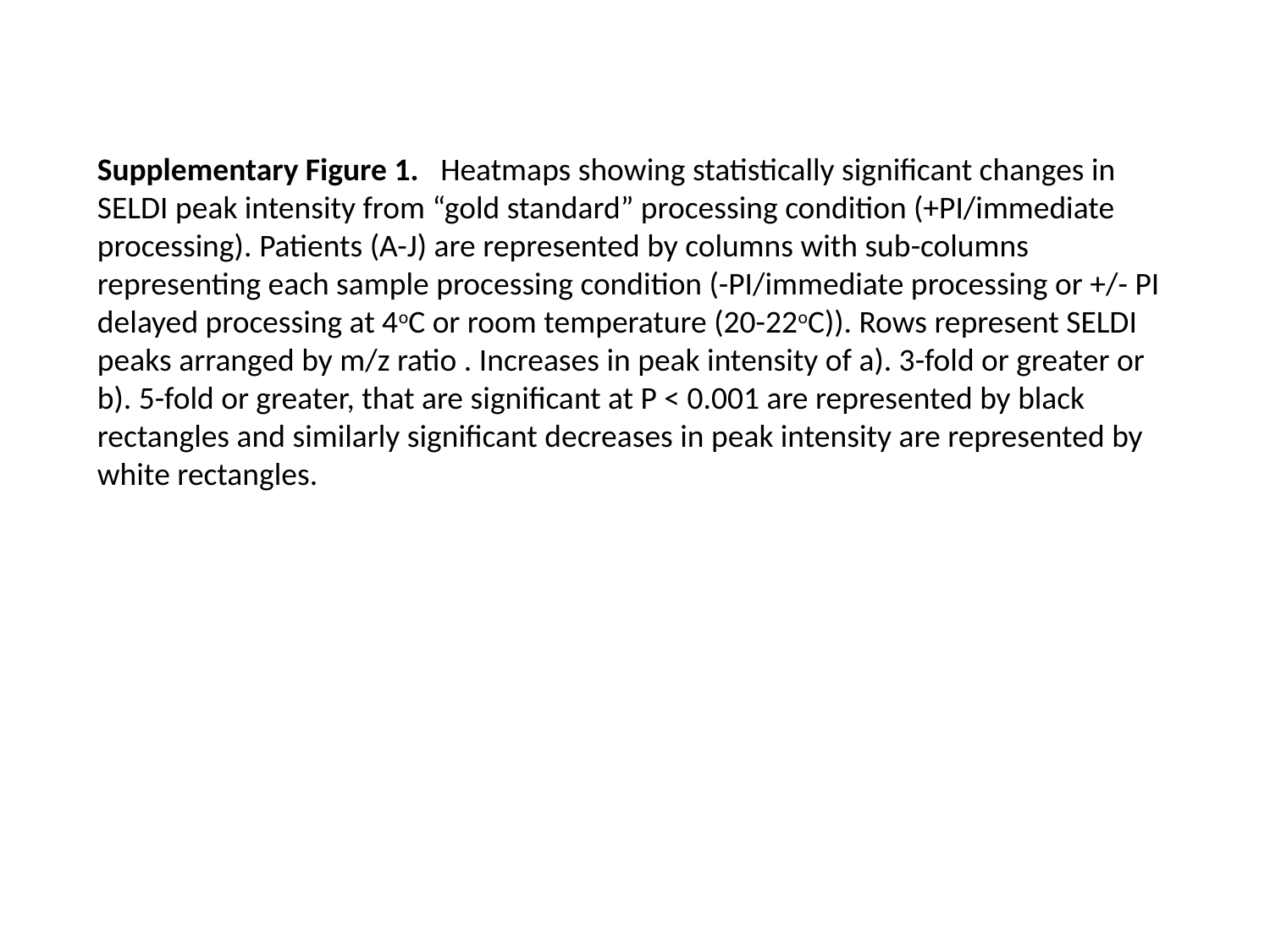

Supplementary Figure 1.   Heatmaps showing statistically significant changes in SELDI peak intensity from “gold standard” processing condition (+PI/immediate processing). Patients (A-J) are represented by columns with sub-columns representing each sample processing condition (-PI/immediate processing or +/- PI delayed processing at 4oC or room temperature (20-22oC)). Rows represent SELDI peaks arranged by m/z ratio . Increases in peak intensity of a). 3-fold or greater or b). 5-fold or greater, that are significant at P < 0.001 are represented by black rectangles and similarly significant decreases in peak intensity are represented by white rectangles.

## Slide 2
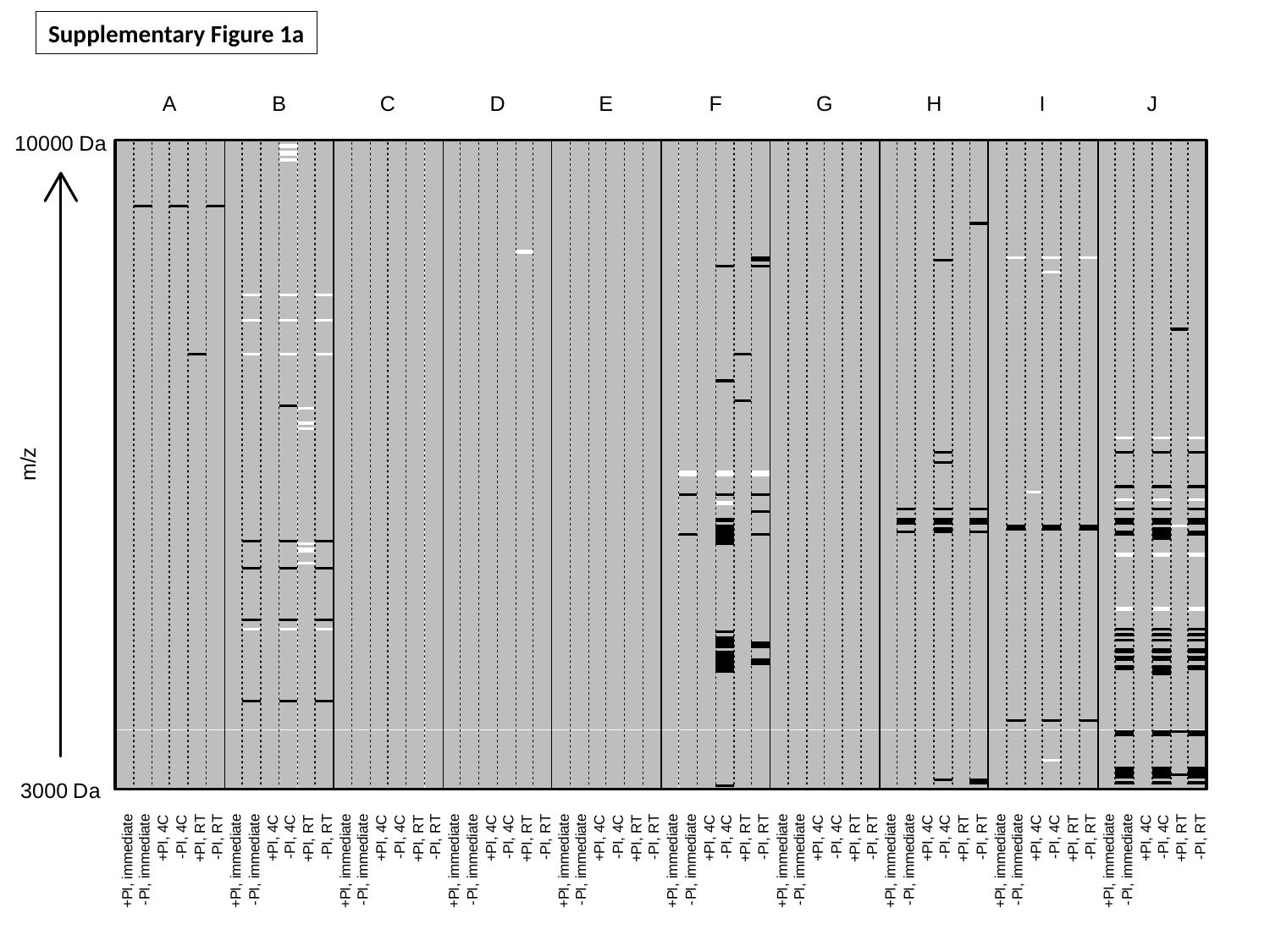

Supplementary Figure 1a

## Slide 3
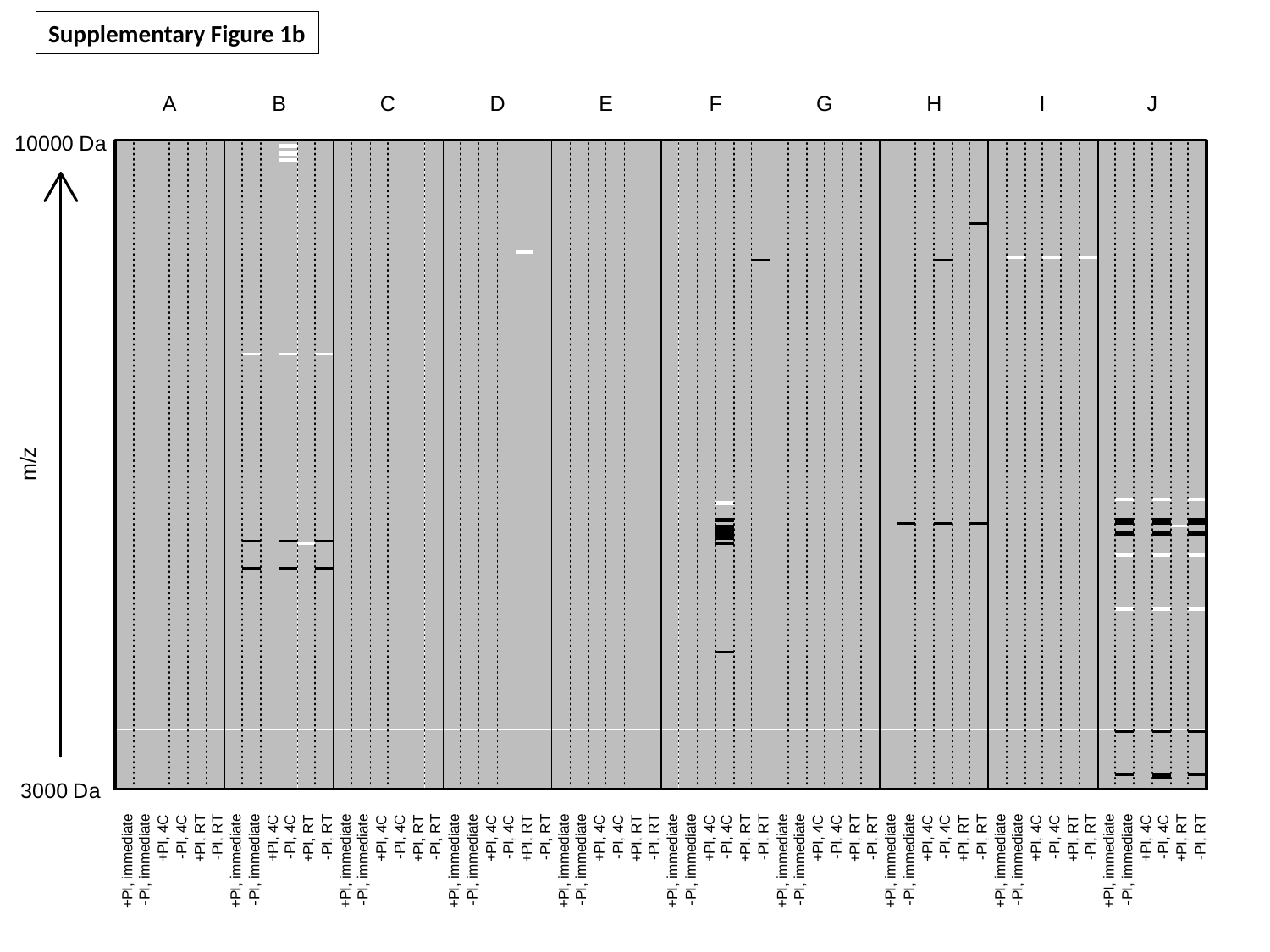

Supplementary Figure 1b
