# Supplementary material for: An analysis of the impact of pre‐analytical factors on the urine proteome: Sample processing time, temperature, and proteolysis
Source: Proteomics Clin Appl. 2015 Feb 26;9(5-6):507–21. doi: 10.1002/prca.201400079 (PMC4964914; doi:10.1002/prca.201400079)
Supplement: Supplementary file 2 — Table S1. Urinary concentrations of α1‐microglobulin (α1M), β2‐microglobulin (β2M), albumin and IgG in 10 different urine samples (A to J) processed in the presence or absence of protease inhibitors either immediately or after delays of 6 hours at 20°C or 4°C. [file PRCA-9-507-s002.docx]

**Supplementary Table 1.** Urinary concentrations of α_1_-microglobulin (α_1_M), β_2_-microglobulin (β_2_M), albumin and IgG in 10 different urine samples (A to J) processed in the presence or absence of protease inhibitors either immediately or after delays of 6 hours at 20^o^C or 4^o^C.

| **Patient** | **Sample processing** | **α_1_M (mg/L)** | **β_2_M (mg/L)** | **Albumin (mg/L)** | **IgG (mg/L)** |
| --- | --- | --- | --- | --- | --- |
| A | +PI/Immed | 19 | 0.2 | 43 | 8 |
|  | -PI/Immed | 20 | 0.1 | 43 | 7 |
|  | +PI/6 hrs 4^o^C | 18 | 0.2 | 44 | 8 |
|  | -PI/6 hrs 4^o^C | 17 | 0.1 | 40 | 7 |
|  | +PI/6 hrs 20^o^C | 18 | 0.1 | 47 | 8 |
|  | -PI/6 hrs 20^o^C | 18 | 0.1 | 36 | 7 |
| B | +PI/Immed | 72 | 1 | 2849 | 113 |
|  | -PI/Immed | 74 | 0.9 | 2873 | 114 |
|  | +PI/6 hrs 4^o^C | 71 | 0.9 | 2727 | 111 |
|  | -PI/6 hrs 4^o^C | 75 | 0.9 | 2801 | 115 |
|  | +PI/6 hrs 20^o^C | 73 | 0.7 | 2810 | 117 |
|  | -PI/6 hrs 20^o^C | 75 | 0.7 | 2808 | 115 |
| C | +PI/Immed | 12 | 2.4 | <3 | 2 |
|  | -PI/Immed | 11 | 2.2 | <3 | 2 |
|  | +PI/6 hrs 4^o^C | 13 | 2.5 | <3 | 2 |
|  | -PI/6 hrs 4^o^C | 11 | 2.3 | <3 | 2 |
|  | +PI/6 hrs 20^o^C | 14 | 2.5 | <3 | 2 |
|  | -PI/6 hrs 20^o^C | 11 | 2.3 | <3 | 2 |
| D | +PI/Immed | 51 | 0.3 | 304 | 36 |
|  | -PI/Immed | 54 | 0.3 | 322 | 39 |
|  | +PI/6 hrs 4^o^C | 52 | 0.3 | 316 | 37 |
|  | -PI/6 hrs 4^o^C | 52 | 0.3 | 314 | 36 |
|  | +PI/6 hrs 20^o^C | 53 | 0.2 | 316 | 37 |
|  | -PI/6 hrs 20^o^C | 52 | 0.3 | 315 | 36 |
| E | +PI/Immed | 27 | 0.9 | 73 | 12 |
|  | -PI/Immed | 26 | 0.9 | 70 | 12 |
|  | +PI/6 hrs 4^o^C | 27 | 0.9 | 73 | 12 |
|  | -PI/6 hrs 4^o^C | 26 | 0.9 | 72 | 11 |
|  | +PI/6 hrs 20^o^C | 27 | 0.8 | 75 | 13 |
|  | -PI/6 hrs 20^o^C | 27 | 0.8 | 75 | 11 |
| F | +PI/Immed | 8 | 0.2 | <20 | 5 |
|  | -PI/Immed | 8 | 0.1 | <20 | 5 |
|  | +PI/6 hrs 4^o^C | 8 | 0.1 | <20 | 7 |
|  | -PI/6 hrs 4^o^C | 7 | 0.1 | <20 | 6 |
|  | +PI/6 hrs 20^o^C | 8 | 0.1 | <20 | 5 |
|  | -PI/6 hrs 20^o^C | 7 | 0.1 | <20 | 4 |
| G | +PI/Immed | 11 | 0.5 | 474 | 48 |
|  | -PI/Immed | 11 | 0.5 | 478 | 48 |
|  | +PI/6 hrs 4^o^C | 11 | 0.5 | 479 | 48 |
|  | -PI/6 hrs 4^o^C | 11 | 0.5 | 484 | 48 |
|  | +PI/6 hrs 20^o^C | 12 | 0.5 | 486 | 49 |
|  | -PI/6 hrs 20^o^C | 11 | 0.5 | 481 | 48 |
| H | +PI/Immed | 6 | 0.1 | <20 | 2 |
|  | -PI/Immed | 6 | <0.1 | <20 | 2 |
|  | +PI/6 hrs 4^o^C | <6 | <0.1 | <20 | 2 |
|  | -PI/6 hrs 4^o^C | <6 | 0.1 | <20 | 2 |
|  | +PI/6 hrs 20^o^C | <6 | <0.1 | <20 | 2 |
|  | -PI/6 hrs 20^o^C | <6 | <0.1 | <20 | 2 |
| I | +PI/Immed | 18 | 1.5 | 42 | 18 |
|  | -PI/Immed | 17 | 1.5 | 36 | 17 |
|  | +PI/6 hrs 4^o^C | 18 | 1.5 | 46 | 19 |
|  | -PI/6 hrs 4^o^C | 17 | 1.5 | 46 | 18 |
|  | +PI/6 hrs 20^o^C | 17 | 1.5 | 46 | 17 |
|  | -PI/6 hrs 20^o^C | 17 | 1.5 | 36 | 16 |
| J | +PI/Immed | <6 | <0.1 | <20 | 7 |
|  | -PI/Immed | <6 | <0.1 | <20 | 7 |
|  | +PI/6 hrs 4^o^C | <6 | <0.1 | <20 | 7 |
|  | -PI/6 hrs 4^o^C | <6 | <0.1 | <20 | 7 |
|  | +PI/6 hrs 20^o^C | <6 | <0.1 | <20 | 6 |
|  | -PI/6 hrs 20^o^C | <6 | <0.1 | - | 7 |
